# Astrocyte Senescence as a Component of Alzheimer’s Disease

**DOI:** 10.1371/journal.pone.0045069

**Published:** 2012-09-12

**Authors:** Rekha Bhat, Elizabeth P. Crowe, Alessandro Bitto, Michelle Moh, Christos D. Katsetos, Fernando U. Garcia, Frederick Bradley Johnson, John Q. Trojanowski, Christian Sell, Claudio Torres

**Affiliations:** 1 Department of Pathology and Laboratory Medicine, Drexel University College of Medicine, Philadelphia, Pennsylvania, United States of America; 2 Department of Pathology and Laboratory Medicine, University of Pennsylvania, Philadelphia, Pennsylvania, United States of America; University of Nebraska Medical Center, United States of America

## Abstract

Aging is the main risk factor for Alzheimer’s disease (AD); however, the aspects of the aging process that predispose the brain to the development of AD are largely unknown. Astrocytes perform a myriad of functions in the central nervous system to maintain homeostasis and support neuronal function. *In vitro*, human astrocytes are highly sensitive to oxidative stress and trigger a senescence program when faced with multiple types of stress. In order to determine whether senescent astrocytes appear *in vivo*, brain tissue from aged individuals and patients with AD was examined for the presence of senescent astrocytes using p16^INK4a^ and matrix metalloproteinase-1 (MMP-1) expression as markers of senescence. Compared with fetal tissue samples (*n* = 4), a significant increase in p16^INK4a^-positive astrocytes was observed in subjects aged 35 to 50 years (*n* = 6; *P* = 0.02) and 78 to 90 years (*n* = 11; *P*<10^−6^). In addition, the frontal cortex of AD patients (*n* = 15) harbored a significantly greater burden of p16^INK4a^-positive astrocytes compared with non-AD adult control subjects of similar ages (*n* = 25; *P* = 0.02) and fetal controls (*n* = 4; *P*<10^−7^). Consistent with the senescent nature of the p16^INK4a^-positive astrocytes, increased metalloproteinase MMP-1 correlated with p16^INK4a^. *In vitro*, beta-amyloid 1–42 (Aβ_1–42_) triggered senescence, driving the expression of p16^INK4a^ and senescence-associated beta-galactosidase. In addition, we found that senescent astrocytes produce a number of inflammatory cytokines including interleukin-6 (IL-6), which seems to be regulated by p38MAPK. We propose that an accumulation of p16^INK4a^-positive senescent astrocytes may link increased age and increased risk for sporadic AD.

## Introduction

Alzheimer’s disease (AD) is the most common cause of dementia, accounting for 60–80% of all cases [1]. The disease is characterized by brain atrophy, extracellular deposition of beta-amyloid (Aβ) peptide, intra-neuronal accumulation of phosphorylated tau, neuronal and synaptic loss, and inflammation. Despite being the focus of intense research, the cause of AD, especially sporadic AD, is still unclear. The greatest risk factor for AD is aging, although the mechanism underlying the contribution of aging to the development AD is poorly understood.

A recent development in the basic biology of aging, with possible implications for AD, is the recognition that senescent cells accumulate *in vivo* [2]–[4]. Although senescent cells increase with age in several tissues [5], little is known about the potential appearance of senescent cells in the brain. The senescence process is an irreversible growth arrest that can be triggered by various events including telomere dysfunction, DNA damage, oxidative stress, and oncogene activation [6]. Although it was once thought that senescent cells simply lack function, it is now known that senescent cells are functionally altered. They secrete cytokines and proteases that profoundly affect neighboring cells, and may contribute to age-related declines in organ function [7]. For example, senescent fibroblasts produce an altered secretory pattern referred to as the senescence-associated secretory phenotype (SASP) [8]. The SASP is characterized by the expression of a complex mixture of factors including reactive cytokines and proteases that create a proinflammatory microenvironment. The relevance of senescent cells to the aging process has been demonstrated by the phenotypic improvement observed following the targeted removal of senescent cells in a progeroid mouse model [9].

Astrocytes comprise a highly abundant population of glial cells, the function of which is critical for the support of neuronal homeostasis. Astrocytes regulate the contents of the synaptic cleft and synaptic transmission as part of the tripartite synapse, control CNS metabolism, and maintain blood brain barrier integrity [10], [11]. Impairment of these functions through any disturbance in astrocyte integrity is likely to impact multiple aspects of brain physiology. Interestingly, astrocytes undergo a functional decline with age *in vivo* that parallels functional declines *in vitro* [12]. We demonstrated that in response to oxidative stress and exhaustive replication, human astrocytes activate a senescence program accompanied by the expression of p16, p21, p53, 53BP1; G1 cell cycle arrest; a reduction in telomere length; and increased co-localization of the histone chaperone HIRA and the promyelocytic leukemia PML proteins, a requirement for the formation of senescence-associated heterochromatin foci [13].

The importance of senescent astrocytes in age-related dementia has been the subject of recent discussion [14], but to date, there is little evidence to suggest that senescent astrocytes accumulate in the brain. In this study, we examined brain tissue from aged individuals and patients with AD to determine whether senescent astrocytes are present in these individuals. Our results demonstrate that senescent astrocytes accumulate in aged brain, and further, in brain from patients with AD. Furthermore, since Aβ peptides induce mitochondrial dysfunction, oxidative stress, and alterations in the metabolic phenotype of astrocytes [15]–[17]; we examined whether Aβ peptides initiate the senescence response in these cells. *In vitro*, we found that exposure of astrocytes to Aβ_1–42_ triggers senescence and that senescent astrocytes produce high quantities of interleukin-6 (IL-6), a cytokine known to be increased in the CNS of AD patients [18]. Based on this evidence, we propose that accumulation of senescent astrocytes may be one age-related risk factor for sporadic AD.

## Results

### Aβ_1-42_ Peptide Leads to Astrocyte Senescence

**Figure 1:**
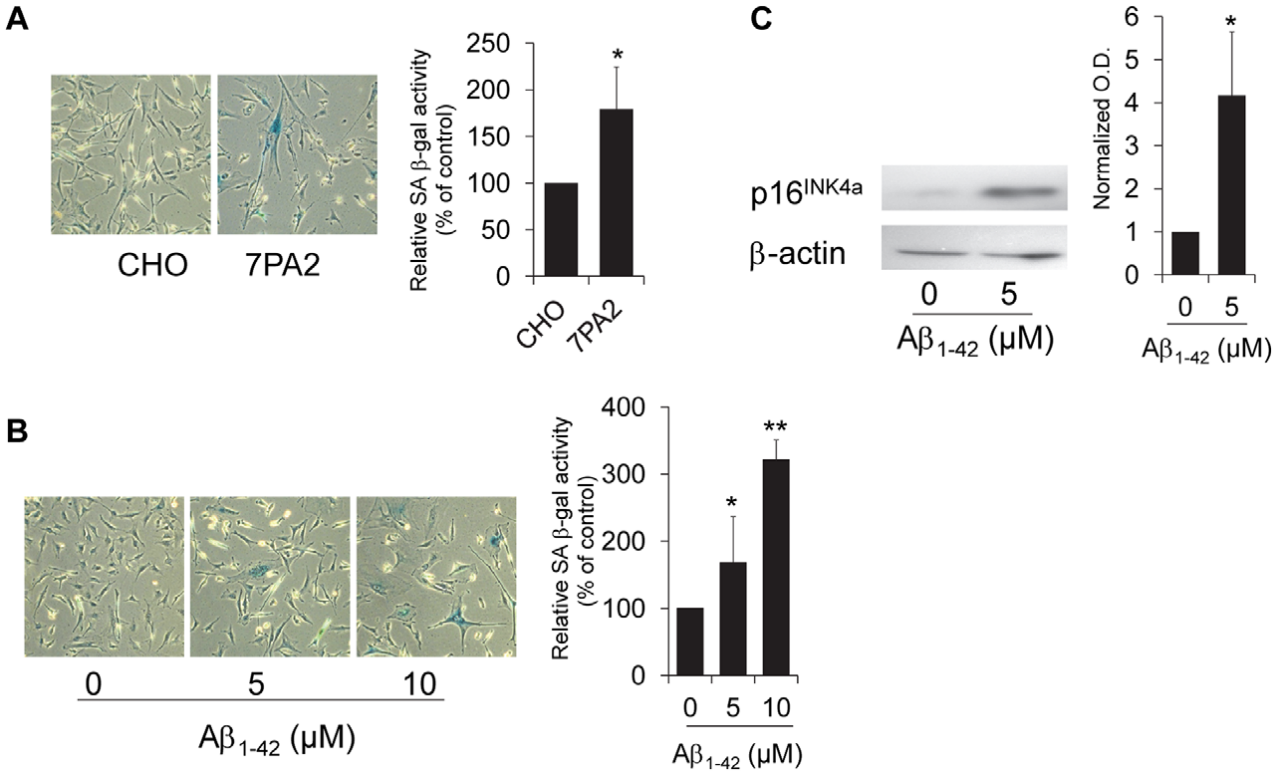
Astrocytes trigger senescence in response to Aβ_1–42_. (**A**) Representative images of astrocytes treated with conditioned media from 7PA2 Aβ-secreting Chinese Hamster Ovary (CHO) cells or CHO control cells as described in Figure S2. Graph shows relative amount of SA β-gal-positive cells from 3 independent experiments, *P = 0.039. (**B**) Representative images of astrocytes treated with oligomerized synthetic Aβ_1–42_ for 2 hours and assayed for SA β-gal activity after 3 days. Graph shows relative levels of SAβ-gal–positive cells from 3 independent experiments, *P = 0.017, Aβ_1–42_ (5 µM and **P = 0.009, Aβ_1–42_ (10 µM) vs. control. (**C**) Immunoblot for p16^INK4a^ expression in astrocytes treated with 5 µM Aβ_1–42_ for 24 hours. Lysate was collected 4 days after treatment initiation. β-actin serves as a loading control. Graph depicts normalized optical density of the ratio of p16^INK4a^:β-actin, *P = 0.02. For all experiments, error bars represent SD and Student’s *t*-test was used to determine significance.

The mechanisms of Aβ-induced neurotoxicity are not fully understood; however, recent studies suggest a role for nonfibrillar soluble oligomers of Aβ in the deleterious effects of Aβ on synaptic plasticity and learned behavior [19]–[22]. Human astrocytes are highly susceptible to stress and undergo a senescent arrest following cellular damage [13], and in order to determine whether a role exists for soluble Aβ_1–42_ in the initiation of senescence in human astrocytes *in vitro*, we treated cells with either conditioned media from Aβ-secreting CHO cells or with synthetic oligomerized Aβ_1–42_ peptide and evaluated markers of cellular senescence. Treatment with either Aβ conditioned media or Aβ_1–42_ peptide induced senescence-associated beta-galactosidase (SA β-gal) activity (Figures 1A, 1B) and increased expression of the p16^INK4a^ senescence biomarker (Figure 1C).

### Senescent Astrocytes Display Characteristics of Senescence-associated Secretory Phenotype (SASP) *in vitro*

**Figure 2:**
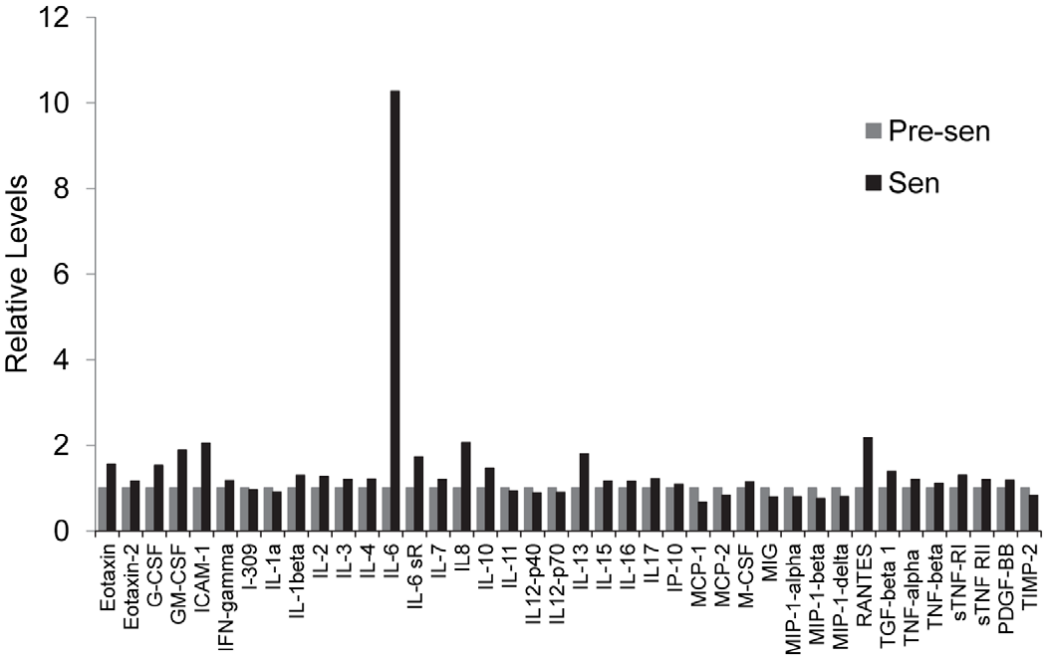
Senescent astrocytes secrete pro-inflammatory mediators that may constitute a SASP. Pre-senescent and senescent astrocytes were incubated in serum-free MCDB105 media. Conditioned media was collected after 48 hours and analyzed by antibody array (RayBiotech, Inc.; Norcross, GA) for pro-inflammatory factors as described in Materials and Methods.

Due to the potential impact of senescent cells on tissue microenvironment, it is important to characterize the secretory pattern that is produced during senescence. We examined the secretory pattern of senescent astrocytes using a protein expression array. This analysis revealed an elevated production of multiple inflammatory cytokines by senescent astrocytes. The most dramatic increase was observed in IL-6 production, which increased 10-fold. Production of RANTES, IL-8, and ICAM-1 was elevated at least 2-fold relative to pre-senescent astrocytes (Figure 2). Increased cytokine production was specific to a subset of cytokines; for example, IL-2, IL-3, and IL-4 showed little to no induction relative to IL-6. One component of the SASP present in multiple cell types is IL-6. IL-6 may act in concert with other pro-inflammatory cytokines to induce local inflammatory responses including the recruitment of natural killer cells to aid in the clearance of senescent cells [14]. In addition to its association with fibroblast senescence [8], [23], IL-6 is thought to be a major mediator of chronic inflammation associated with aging and aging-related diseases including AD [24]–[26].

### The population of senescent astrocytes increases in human brain during aging and AD

**Figure 3:**
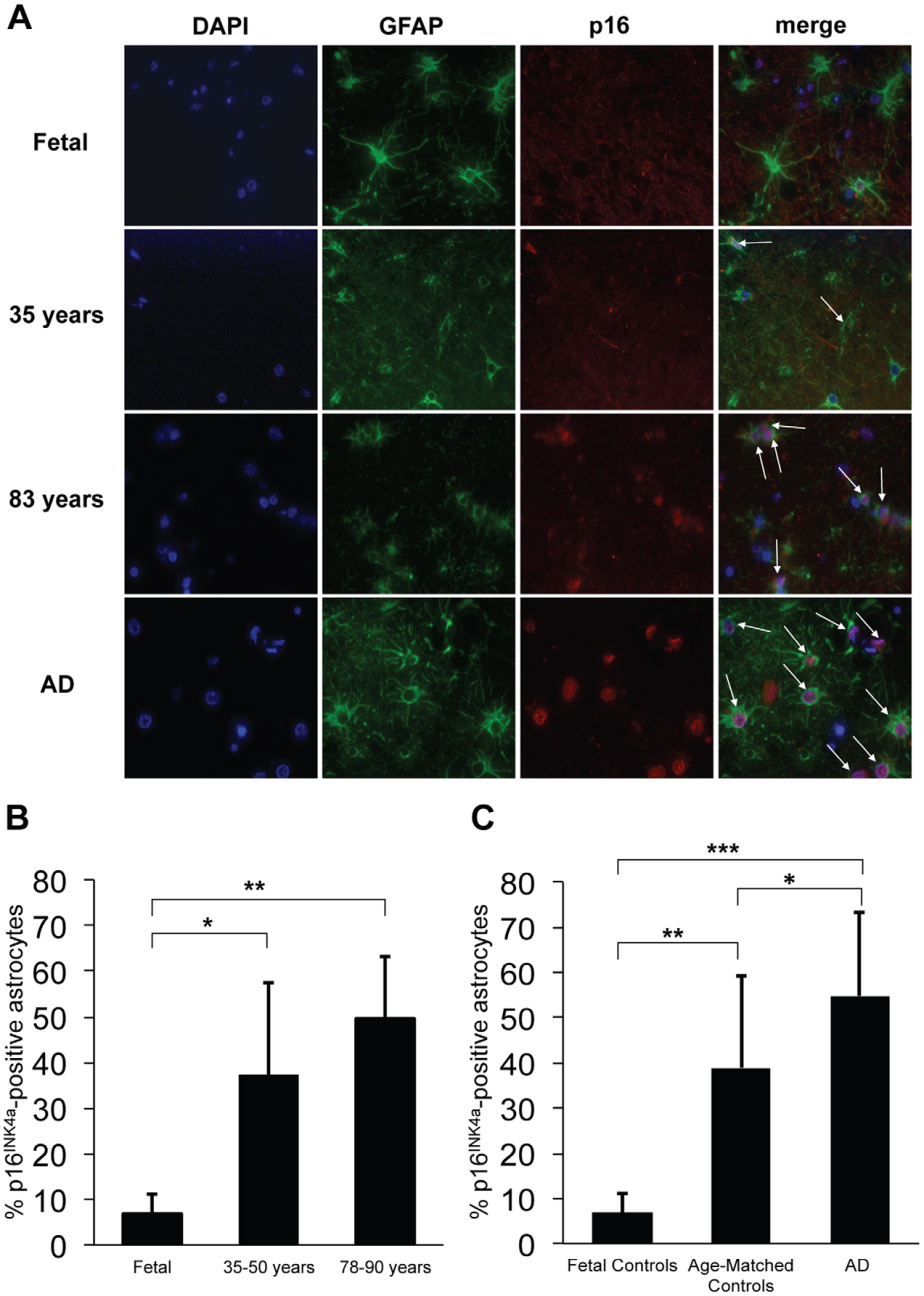
Increased frequency of senescent astrocytes during brain aging and AD. p16^INK4a^-positive astrocytes were identified in formalin-fixed, paraffin-embedded frontal cortex sections by double immunofluorescence with p16^INK4a^/GFAP. (**A**) Double immunofluorescence with p16^INK4a^/GFAP showing increased p16^INK4a^-positive astrocytes with increased age and AD (representative images). Blue: DAPI; green: GFAP; red: p16^INK4a^. Arrows indicate p16^INK4a^-positive astrocytes. (**B**) Bar diagram shows increased mean p16^ INK4a^-positive astrocytes in frontal cortices from non-AD adult subjects (35–50 years, *n* = 6; 78–90 years, *n* = 11) as compared to fetal autopsy tissue (*n* = 4). *P = 0.02, fetal vs. 35–50 year olds; **P<10^−6^, fetal vs. 78–90 year olds; Student’s *t*-test. (**C**) Bar diagram shows increased mean p16^ INK4a^-positive astrocytes in frontal cortices from AD adult subjects (*n* = 15) compared to non-AD adult control subjects (*n* = 25) of similar ages; and fetal controls (*n* = 4). ^*^ *P* = 0.02, AD vs. adult controls; ***P<10^−7^, AD vs. fetal controls, and **P<10^−6^, adult controls vs. fetal controls. Error bars represent SD and Student’s *t*-test was used to determine significance. At least 200 cells were counted per slide.

**Figure 4:**
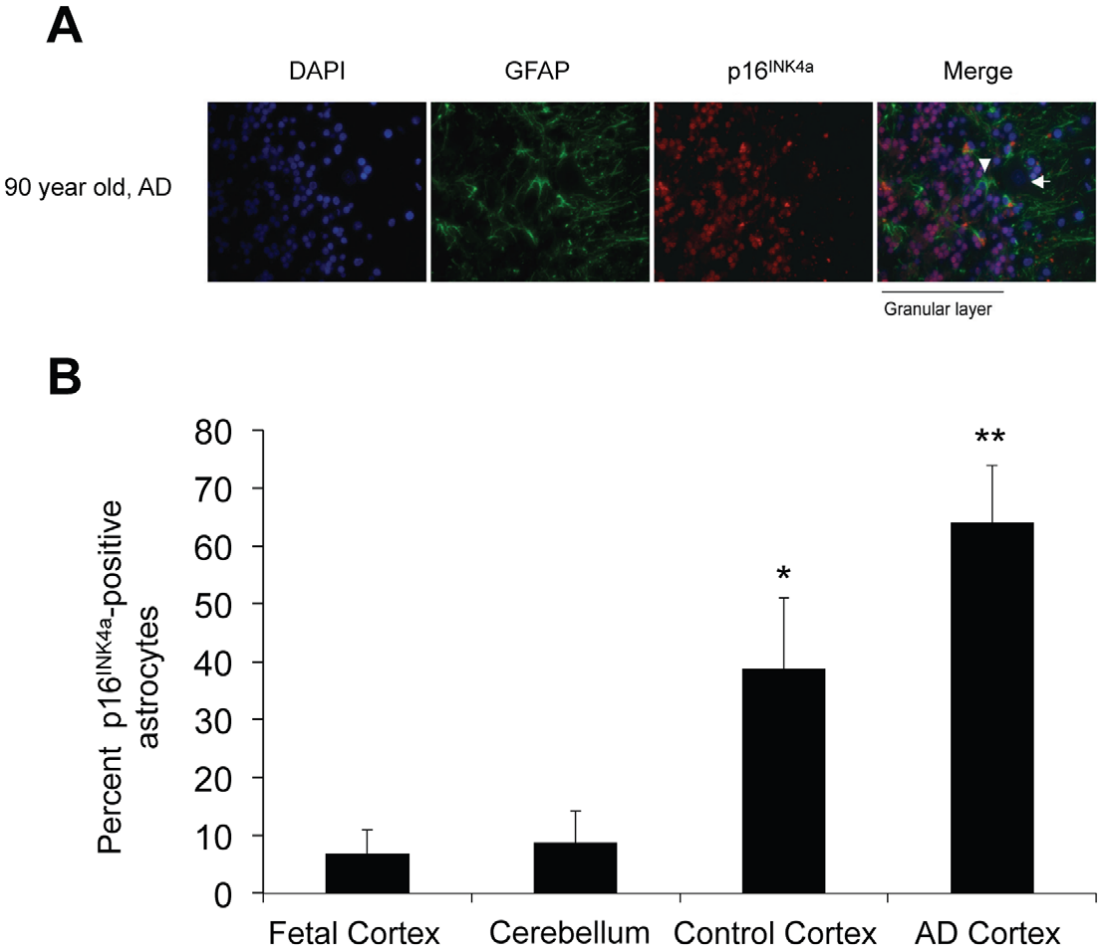
Expression level of p16^INK4a^ is not elevated in adult cerebellar astrocytes. (**A**) Representative images of cerebellum from an AD patient showing intensely p16^INK4a^ positive granular layer. The Bergmann glia (arrowhead) and Purkinje neurons (arrow) are negative. Blue: DAPI; Green: GFAP; Red: p16^INK4a^. (**B**) Bar diagram shows increased mean percentage of p16^INK4a^-positive astrocytes in formalin-fixed, paraffin-embedded cortical sections from AD subjects (n = 3) and control subjects (n = 3) compared to cerebellum from both AD and aged-matched controls (n = 6). The mean percentage of p16^INK4a^-positive astrocytes in cerebellum is comparable to fetal cortical levels. *P = 0.04, Student’s t test, cerebellum vs. control cortices; **P = 0.004, Student’s t test, cerebellum vs. AD cortices. Error bars represent SD At least 200 cells per slide were counted for the cortical sections, and at least 100 cells per slide were counted for the cerebellar sections.

Having established that Aβ can induce senescence *in vitro*, we examined brain tissue from patients of differing ages and those with AD (See Table S1 for patient demographics and clinical history) for p16^INK4a^ expression. p16^INK4a^ has emerged as a robust *in vivo* biomarker of senescence in human and rodent tissues [27], [28]. Frontal cortices of 44 cases (4 fetal and 40 adult) were screened for p16^INK4a^-positive astrocytes using double immunofluorescence for p16^INK4a^ and the astrocyte marker GFAP (Figure 3A). Although the average age at initial presentation of AD ranges from 68–73 years across different ethnicities, neuropathologic changes characteristic of AD can precede clinical symptoms by up to twenty years [29], [30]. We selected the age ranges of 35–50 years and 78–90 years (Figure 3B) in order to compare a group that is least likely to have AD and a group that is most likely to have AD [31]. Normal adults exhibited a 6- to 8-fold increase in the number of astrocytes expressing p16^INK4a^ compared with fetal cortices, suggesting an accumulation of p16^INK4a^-positive astrocytes with aging (Figure 3B). This is consistent with reports indicating similar *in vivo* increases in p16^INK4a^ in human aged skin [27], kidney [32], lung [33], and heart [34]. Frontal cortices from AD patients demonstrated a significant increase in p16^INK4a^ expression when compared to age-matched controls (Figure 3C). Overall, there was an increase in p16^INK4a^-positive astrocytes from fetal to non-AD adults and from non-AD to AD adults. This suggests that senescent astrocytes do accumulate with normal aging, and increase further in the setting of AD. Cerebellar astrocytes (AD and similar-aged controls) failed to demonstrate an increase in p16^INK4a^ expression (Figure 4). This finding is consistent with a diffuse cerebellar amyloid plaque formation and relative lack of cerebellar pathology in AD [35], [36].

**Figure 5:**
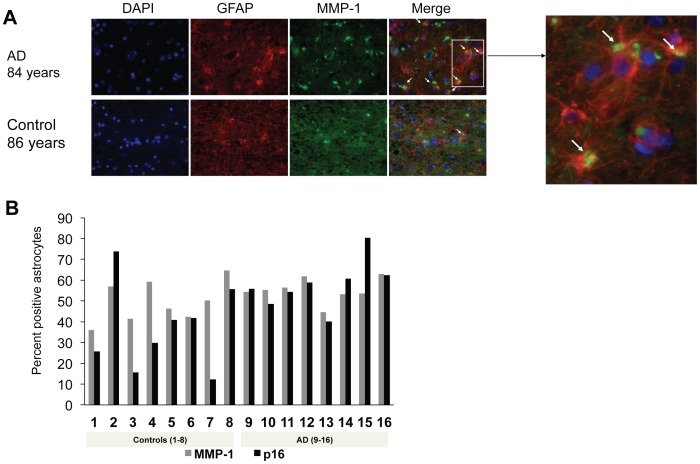
MMP-1 expression parallels p16^INK4a^ expression in human astrocytes in vivo. (**A**) Double immunofluorescence with MMP-1/GFAP in AD and a similar-aged control (representative images). Blue: DAPI; red: GFAP; green: MMP-1. Arrows indicate MMP-1–positive astrocytes. Inset shows magnified image of MMP-1-positive astrocytes (yellow: cytoplasmic). (**B**) Bar diagram shows positive correlation between MMP-1 and p16^INK4a^ expression in human frontal cortex, independent of diagnosis, *P* = 0.02, Spearman’s correlation coefficient = 0.574.

Increased secretion of matrix metalloproteinase-1 (MMP-1) during senescence of human fibroblasts may affect the remodeling of the extracellular matrix and tissue function during aging [37]. Consistent with the senescent nature of the p16^INK4a^-positive astrocytes, increased MMP-1/collagenase correlated with p16^INK4a^ during brain aging, suggesting that senescent astrocytes may have an altered pattern of secretion *in vivo* with potential consequences for the microenvironment (Figure 5A). As shown in Figure 5B, p16^INK4a^ and MMP-1 staining in astrocytes demonstrated a positive correlation with each other (Spearman correlation coefficient 0.574, *P* = 0.02). Comparison of MMP-1 expression based on the presence or absence of comorbidities did not reveal any significant differences (data not shown).

### p38MAPK is Activated in Senescent Astrocytes and is Required for Generation of the SASP

**Figure 6:**
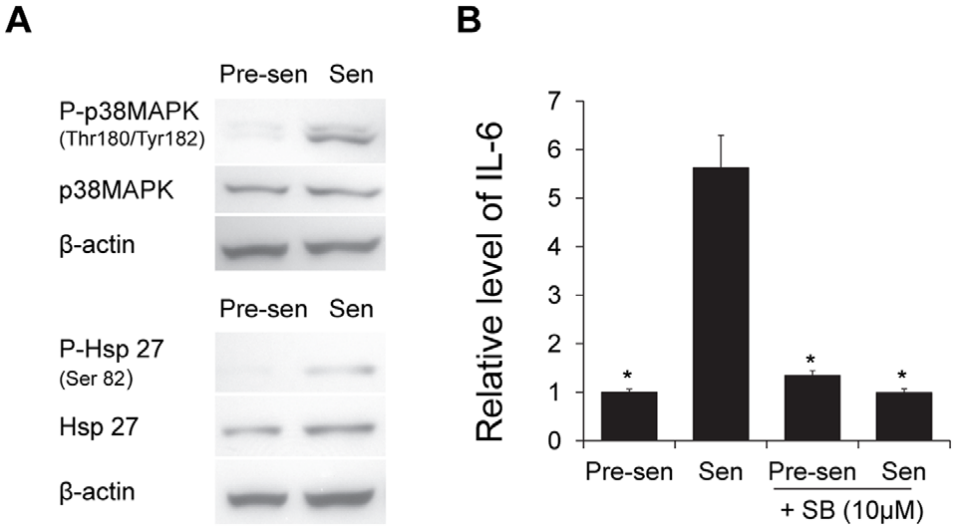
p38MAPK pathway is activated and modulates IL-6 secretion in astrocytes. (**A**) Western blots depict the levels of total and phosphorylated p38MAPK and heat shock protein 27 (Hsp27) in pre-senescent and H_2_O_2_-induced senescent astrocytes with β-actin as loading control. (**B**) Pre-senescent and senescent astrocytes were treated with 10 µM SB-203580 or DMSO as a control for 48 hours prior to incubation in serum-free MCDB105 media. Conditioned media was collected after 24 hours and IL-6 was analyzed by ELISA (R&D Systems, Inc.; Minneapolis, MN) and normalized to cell number. Graph depicts the relative level of IL-6 (n = 3), *P<0.01 vs. senescent, Student’s t test.

Whereas p16^INK4a^ is a robust biomarker for detecting senescent astrocytes both *in vitro* and in tissues, elevated expression of this cyclin-dependent kinase inhibitor is not essential for the generation of a SASP [38]. Therefore, we examined senescent astrocytes for activation of p38 mitogen-activated protein kinase (p38MAPK), which is a mediator of the senescence arrest in response to diverse stimuli [39] and a known regulator of the SASP in other cell types [40]. An increase in p38MAPK signaling has also been linked to the cognitive decline associated with AD pathophysiology [41], [42]. Activity of this stress-related kinase (assessed by phosphorylation of p38MAPK and heat shock protein 27 [Hsp27], a major downstream target of p38MAPK activation) increased dramatically during senescence in astrocytes (Figure 6A). In addition, pharmacological inhibition of p38MAPK abolished IL-6 secretion by senescent astrocytes (Figure 6B).

## Discussion

Elevated markers of DNA damage have been observed in astrocytes found in regions of the brain that are vulnerable to AD [43]. While a role for senescent astrocytes in the aging brain has been postulated [14], evidence for the existence of these cells has been lacking. Our study provides evidence supporting the existence of senescent cells in the human brain. We demonstrated relatively high numbers of senescent astrocytes in brain tissue from aged individuals and patients suffering from AD. Further studies will be required in order to definitively demonstrate a casual connection between the appearance of senescent astrocytes and CNS dysfunction. Furthermore, the number of cases was limited by the availability of post-mortem samples. This was partly due to the low rate of autopsies in general [44].

Evidence coming mainly from fibroblast models indicates that senescent cells secrete pro-inflammatory mediators and proteases that not only potentiate clearance of senescent cells through immune surveillance, but may also contribute to age-related declines in organ function [7], [8], [45]. Consistent with these observations, we found that the accumulation of p16^INK4a^-positive astrocytes is accompanied by an increase in MMP-1/collagenase, a metalloproteinase able to degrade collagen types I, II, and III, at the extracellular matrix. In agreement with these results, increased expression of MMP-1 has been reported in normal senescent human and Werner fibroblasts [37], [46], [47]. Additionally, our *in vitro* data indicate an altered pattern of secretion in senescent astrocytes that is characterized by significant increases in IL-6 production. We propose a key role for p38MAPK in the regulation of IL-6 in these cells. Overall, these results provide support for a SASP in human astrocytes during aging *in vivo*. Several inflammatory markers found to be robustly changed in other cell types were not as dramatically altered in senescent (replicative) astrocytes according to our array [25]. Potential reasons for this discrepancy are the nature of senescence induction in the astrocytes and cell-type specific differences. Furthermore, only a subset of factors indicative of the SASP was measured in the antibody array. Further analysis will be required to determine whether the full breadth of SASP factors is altered in the secretion pattern of senescent astrocytes.

The data suggest that several factors may contribute to the appearance of p16^INK4a^-positive cells. The presence of comorbidities such as diabetes, hypertension, or chronic obstructive pulmonary disease correlated with the burden of p16^INK4a^-positive astrocytes. Also within our cohort of patients, a subset of patients exists with documented history of severe hypoxic episodes or cerebral ischemia. These patients show high positive staining for p16^INK4a^. This effect was most pronounced in a 49-year-old male AD patient with a family history of AD and neuropathologic evidence of hypoxia. Interestingly, similar p16^INK4a^ levels were observed in a non-AD patient with a clinical history of repeated hypotensive and hypoxic events due to cardiac failure. Although patient numbers are too small to perform a statistical evaluation of the relationship between clinical history and the appearance of senescent astrocytes, we propose that the number of p16^INK4a^-positive astrocytes is a reflection of the cumulative impact of multiple stressors resulting from ischemic events, focal areas of cellular damage secondary to ROS production, Aβ deposition, or other insults. The fact that senescence is a common response to multiple injuries or genetic abnormalities supports the concept that the accumulation of p16^INK4a^-positive senescent astrocytes may link aging as a risk factor for sporadic AD. Interestingly, senescence in the CNS may not be exclusively limited to astrocytes. We observed nuclei that were positive for p16^INK4a^, but not GFAP, in the frontal cortex (data not shown). Senescence in other cell types of the CNS, including neurons, has been postulated as important risk factor for AD [48].

*In vitro*, the exposure of astrocytes to oligomeric species of Aβ_1–42_ peptides or cell-derived Aβ induces astrocyte senescence as measured by several markers of the senescent arrest. Similarly, Aβ peptides accelerate the senescence of endothelial cells *in vitro* and *in vivo* in the zebrafish model [49]. The level of soluble Aβ oligomers in AD brain correlates better with disease severity as compared with the level insoluble fibrillar Aβ [50]; therefore, we chose to use Aβ oligomers at concentrations previously shown to affect astrocytes [51], [52]. Compared with cell-derived Aβ, a considerably higher concentration of synthetic Aβ_1–42_ peptide, micromolar and nanomolar respectively, was required to induce senescence in cultured human astrocytes. The increased dosage of synthetic versus cell-derived Aβ oligomers required to trigger senescence, correlates with reports on the effects of Aβ on rat hippocampal long-term potentiation [53].

Thus, it appears that the cellular stress induced by Aβ results in activation of the senescence program. In fact, astrocytes are very sensitive to stress, rapidly triggering the senescent phenotype in response to a mild oxidative stress [13].Consistent with this concept, we have demonstrated that astrocytes cultured at 7% oxygen have an extension in their replicative life span compared with those cultured at 20% oxygen (Figure S1). Based on these results, and our previously published findings indicating increased sensitivity of astrocytes to oxidative stress [13], we hypothesize that there is a functional relationship between Aβ and senescence, and that Aβ may induce oxidative stress to trigger the senescent program in astrocytes, thereby contributing to astrocyte dysfunction. The chronic, subtle loss of astrocyte ability to maintain homeostasis throughout the CNS is now thought to underlie AD pathogenesis [54].

A common pathological feature of many neurodegenerative disease states, including AD, is reactive astrogliosis, which is a well-characterized spectrum of changes that occurs in astrocytes in response to a variety of insults [55]. It remains to be determined how our finding of stress-induced senescence in astrocytes relates to reactive astrogliosis; however, we propose that these two phenotypes may be interrelated given that astrocytes are highly susceptible to stress-induced changes. Based on the results presented here, we propose that the presence of senescent astrocytes contributes to the pathogenesis of AD and may represent a link between the aging process and progression of the disease.

## Materials and Methods

### Ethics Statement

All tissue samples were de-identified and obtained after approval by the Institutional Review Board at Drexel University College of Medicine (Protocol Number: 18172), and Institutional Review Board at University of Pennsylvania (Protocol Number: 180600). The protocols were approved as an exempt study. An honest broker de-identifed each specimen and no protected health information was made available to the researcher. The specimens were assigned de-identified numbers which could not be traced back to the patients. There is no link between patient identifiers and the data, therefore consent for this protocol was waived.

### Human Frontal Cortex Samples

Archived, formalin-fixed, paraffin-embedded (FFPE) human frontal cortex (n = 44) and cerebellum (n = 6) slides were obtained from the Drexel University College of Medicine Department of Pathology & Laboratory Medicine’s autopsy service and the University of Pennsylvania’s Center for Neurodegenerative Disease Research. Samples were obtained from normal subjects aged 36 weeks to 92 years at autopsy. The fetal samples ranged in estimated gestational age from 36 to 39 weeks. Additional samples from patients with a diagnosis of AD at autopsy were obtained ranging in age from 49 to 90 years. Control subjects were nonschizophrenic subjects without AD.

### Cell Culture

Human astrocytes were cultured at 37°C, 5% CO_2_ in astrocyte medium supplemented with 2% fetal bovine serum, growth supplement, and penicillin/streptomycin all obtained from ScienCell Research Laboratories (Carlsbad, CA). Cells were seeded at a standard density of 1 × 10^4^ cells/cm^2^ and cultured until they reached 70% to 80% confluence. At each passage, astrocytes were trypsinized, counted, and the cumulative population doubling level (CPDL) was calculated as we have previously described [13].

### Preparation of Oligomeric Beta-amyloid

Synthetic Aβ_1–42_ (American Peptide; Sunnyvale, CA) was oligomerized as described [56], [57]. Briefly, lyophilized Aβ_1–42_ was solubilized in hexafluoroisopropanol HFIP (1,1,1,3,3,3-hexafluoro-2-propanol) to obtain monomers. Aβ_1–42_/HFIP aliquots were dried in low-protein binding tubes using a speedvac and the resulting peptide film was stored at −80°C until use. To generate oligomeric Aβ, the peptide film was dissolved in DMSO to a concentration of 5 mM and sonicated. The suspension was diluted in astrocyte medium to achieve the desired final concentrations and incubated at 4°C overnight. The presence of soluble Aβ oligomers in astrocyte medium following oligomerization was verified by immunoblotting of approximately 0.1 µg of synthetic peptide loaded onto a 12% SDS-PAGE gel (Figure S2).

### Preparation of Conditioned Media from 7PA2 and CHO Cells

The 7PA2 cell line (kindly provided by Dr. E.H. Koo; University of California, San Diego) stably expresses the mutant amyloid precursor protein (APP) gene as previously described [58], and secretes Aβ peptides at levels similar to those found in human brains affected by AD and normal cerebrospinal fluid (∼1–2 nM) [59]. The Aβ produced by these cells is biologically relevant as judged by a significant disruption in working memory when delivered to rats [22], [60]. CHO cells, originally derived from Chinese hamster ovary [61], were available and carried in our laboratory. Aβ-secreting 7PA2 or untransfected control CHO cells were grown to near confluence and incubated in serum-free DMEM or MCDB105 media for 24 hours. Conditioned media was collected and cleared of cells by centrifugation at 200 x *g* for 10 min at 4°C. Media was then concentrated 15X using 3,000 MWCO centrifugal filters (Millipore; Cork, Ireland). Concentrated media was stored at −80°C prior to treatment. The presence of soluble Aβ oligomers in conditioned media was verified by immunoblot using anti-Aβ antibody (2C8) (Santa Cruz Biotechnology; Santa Cruz, CA). Levels of Aβ in 7PA2 conditioned media are shown in Figure S2.

### Staining for Senescence-associated β-galactosidase

Senescence-associated beta-galactosidase (SA β-gal) staining was performed as previously described [62]. Briefly, following exposure to Aβ peptides or conditioned media, astrocytes were fixed in 2% formaldehyde/0.2% glutaraldehyde for 5 minutes and stained for SA β-gal activity overnight. The percent of positive (blue) cells were counted and expressed relative to the level of positive cells in vehicle-treated controls. At least 200 cells were counted.

### Analysis of Inflammatory Factors Secreted by Astrocytes

Pre-senescent (early-passage) and senescent (late-passage) human astrocytes were incubated with serum-free MCDB105 media to generate conditioned media. After a 48-hour incubation period, media was collected and cells were trypsinized and counted to determine the cell number for normalization. Human inflammation antibody array (RayBiotech, Inc.; Norcross, GA) was used to detect secreted inflammatory factors present in the conditioned media according to the product manual. The intensity of the signal on the array membranes was quantified by densitometry, normalized to cell number, and analyzed using the RayBio® Analysis Tool.

### Role of p38MAPK in IL-6 Secretion

Human astrocytes were triggered to undergo stress-induced premature senescence by H_2_O_2_ treatment (200 µM for 2 hours). Five days following senescence induction, astrocytes were treated with 10 µM of the p38MAPK pharmacological inhibitor SB-203580 (Enzo Life Sciences Inc.; Farmingdale, NY) or vehicle for 48 hours and then incubated in serum-free MCDB105 for conditioned media collection. Pre-senescent astrocytes were also treated with 10 µM SB-203580 or vehicle in the same manner prior to conditioned media collection. Conditioned media was collected from pre-senescent and senescent cells after a 24-hour incubation period in serum-free MCDB105. The level of IL-6 in conditioned media was quantified by IL-6 ELISA (R&D Systems, Inc.; Minneapolis, MN) and normalized to cell number.

### Immunoblotting

Early-passage human astrocytes were treated for 24 hours with oligomerized Aβ_1–42_ at a final concentration of 5 µM in astrocyte media; 0.05% DMSO was used as control. After 24 hours, media was replaced. Cell lysate was collected 4 days post-treatment in RIPA buffer. Western blot analysis was performed under standard conditions using 25 µg of total cell proteins for anti-p16^INK4a^ (Santa Cruz Biotechnology; Santa Cruz, CA) and anti-β-actin (Sigma Aldrich; St. Louis, MO) as a loading control. In order to detect activation of the p38MAPK signaling pathway, antibodies recognizing p38MAPK and phospho-p38MAPK (Thr180/Tyr182) were obtained from Santa Cruz Biotechnology (Santa Cruz, CA), along with antibodies against Hsp27 (R&D Systems; Minneapolis, MN) and phospho-Hsp27 (Ser82) (Cell Signaling Technology; Danvers, MA).

### Immunofluorescence

Slides were de-paraffinized with xylene, 3×5 minutes. After washes in 90% EtOH, 80% EtOH, 70% EtOH, and de-ionized water (2×5 min each), antigen retrieval was performed by heat-steam for 20 minutes in 10 mM citrate buffer pH 6.0. Slides were blocked in 5% normal goat serum, 5% normal donkey serum, 0.1% BSA, 0.25% Triton X-100 for 1.5 hours, then incubated overnight in a humidified chamber with anti-GFAP (Millipore; Temecula, CA), anti-p16^INK4a^ (Santa Cruz Biotechnology, Inc.; Santa Cruz, CA) and anti-MMP1 (Lifespan Biosciences; Seattle, WA) primary antibodies. Slides were washed 3×5 minutes in 0.1% BSA and 0.1% Triton X-100, then incubated for 1 hour in the dark at room temperature with the secondary antibodies anti-rabbit Alexa Fluor 488 and anti-mouse Alexa Fluor 555 (both Invitrogen; Carlsbad, CA). After 3×5 minute washes in 1X PBS with 0.1% BSA and 0.1% Triton X-100, slides were incubated with DAPI, and mounted with Vectashield Mounting Medium (Vector Laboratories, Inc.; Burlingame, CA). Cells were visualized using an Olympus BX61 fluorescence microscope coupled with a Hamamatsu ORCA-ER camera and using Slide Book 4 software version 4.0.1.44 (Intelligent Innovations, Inc.; Denver, CO). 200-cell counts were performed at 40X magnification for p16^INK4a^-GFAP and MMP1-GFAP.

### Statistics

Unless otherwise specified, all experiments were done at least in triplicate. Data are expressed as mean ± SD.

## Supporting Information

Table S1 **Clinical history obtained from post-mortem reports.** A total of 44 cases (4 fetal and 40 adults) were included in the study. Adult ages ranged from 35 to 92 years (mean 72.3±17.4; 10 males, 15 females) for controls and 49 to 90 years (mean 77.6±11.44; 8 males, 7 females) for patients with AD. The average postmortem interval was 13.31±9.44 hours (N = 36, range 3 to 43.5 hours). Fetal samples were obtained from fetuses that died in utero and therefore had uncertain postmortem intervals. Cortical sections were obtained from the frontal cortex in all the adult cases.
(DOCX)

Figure S1 **Effect of oxygen tension on replicative lifespan.** Astrocytes were grown at 5% CO_2_ and either 20% or 7% oxygen. At every passage, cells were counted and the cumulative population doubling was calculated as described [13].
(TIF)

Figure S2 **Detection of oligomerized Aβ peptide.** Top gel, synthetic amyloid-β peptide (Aβ_1–42_) was diluted in astrocyte media to obtain a final concentration of 1µM and oligomerized as described. Astrocyte media with dimethyl sulfoxide (DMSO) alone was used as a control. A volume of media containing approximately 0.094 µg of synthetic peptide was loaded onto 12% gel. Western blot depicts the presence of small molecular weight oligomers of Aβ in astrocyte media. Bottom gel, conditioned media from 7PA2 cells contains secreted Aβ. 7PA2 and control CHO cells were incubated with serum-free DMEM or MCDB105 media for 24 hours to generate conditioned media. Conditioned media was collected and concentrated as described. Western blot showing presence of Aβ in conditioned media from 7PA2 cells.
(TIF)

## References

[1] Thies W, Bleiler L (2011) 2011 Alzheimer’s disease facts and figures. Alzheimers Dement 7: 208–244. 21414557 10.1016/j.jalz.2011.02.004

[2] Herbig U, Ferreira M, Condel L, Carey D, Sedivy JM (2006) Cellular senescence in aging primates. Science 311: 1257. 16456035 10.1126/science.1122446

[3] Jeyapalan JC, Sedivy JM (2008) Cellular senescence and organismal aging. Mech Ageing Dev 129: 467–474. 18502472 10.1016/j.mad.2008.04.001 PMC3297662

[4] Michaloglou C, Vredeveld LC, Soengas MS, Denoyelle C, Kuilman T, et al (2005) BRAFE600-associated senescence-like cell cycle arrest of human naevi. Nature 436: 720–724. 16079850 10.1038/nature03890

[5] Jeyapalan JC, Ferreira M, Sedivy JM, Herbig U (2007) Accumulation of senescent cells in mitotic tissue of aging primates. Mech Ageing Dev 128: 36–44. 17116315 10.1016/j.mad.2006.11.008 PMC3654105

[6] Rodier F, Campisi J (2011) Four faces of cellular senescence. J Cell Biol 192: 547–556. 21321098 10.1083/jcb.201009094 PMC3044123

[7] Campisi J, Andersen JK, Kapahi P, Melov S (2011) Cellular senescence: a link between cancer and age-related degenerative disease? Semin Cancer Biol 21: 354–359. 21925603 10.1016/j.semcancer.2011.09.001 PMC3230665

[8] Coppe JP, Desprez PY, Krtolica A, Campisi J (2010) The senescence-associated secretory phenotype: the dark side of tumor suppression. Annu Rev Pathol 5: 99–118. 20078217 10.1146/annurev-pathol-121808-102144 PMC4166495

[9] Baker DJ, Wijshake T, Tchkonia T, Lebrasseur NK, Childs BG, et al. (2011) Clearance of p16(Ink4a)-positive senescent cells delays ageing-associated disorders. Nature. 10.1038/nature10600 PMC3468323 22048312

[10] Sofroniew MV, Vinters HV (2010) Astrocytes: biology and pathology. Acta Neuropathol 119: 7–35. 20012068 10.1007/s00401-009-0619-8 PMC2799634

[11] Halassa MM, Fellin T, Haydon PG (2009) Tripartite synapses: roles for astrocytic purines in the control of synaptic physiology and behavior. Neuropharmacology 57: 343–346. 19577581 10.1016/j.neuropharm.2009.06.031 PMC3190118

[12] Pertusa M, Garcia-Matas S, Rodriguez-Farre E, Sanfeliu C, Cristofol R (2007) Astrocytes aged in vitro show a decreased neuroprotective capacity. J Neurochem 101: 794–805. 17250685 10.1111/j.1471-4159.2006.04369.x

[13] Bitto A, Sell C, Crowe E, Lorenzini A, Malaguti M, et al (2010) Stress-induced senescence in human and rodent astrocytes. Exp Cell Res 316: 2961–2968. 20620137 10.1016/j.yexcr.2010.06.021

[14] Salminen A, Ojala J, Kaarniranta K, Haapasalo A, Hiltunen M, et al (2011) Astrocytes in the aging brain express characteristics of senescence-associated secretory phenotype. Eur J Neurosci 34: 3–11. 21649759 10.1111/j.1460-9568.2011.07738.x

[15] Abramov AY, Canevari L, Duchen MR (2004) Beta-amyloid peptides induce mitochondrial dysfunction and oxidative stress in astrocytes and death of neurons through activation of NADPH oxidase. J Neurosci 24: 565–575. 14724257 10.1523/JNEUROSCI.4042-03.2004 PMC6729998

[16] Rhein V, Baysang G, Rao S, Meier F, Bonert A, et al (2009) Amyloid-beta leads to impaired cellular respiration, energy production and mitochondrial electron chain complex activities in human neuroblastoma cells. Cell Mol Neurobiol 29: 1063–1071. 19350381 10.1007/s10571-009-9398-y PMC11506282

[17] Allaman I, Gavillet M, Belanger M, Laroche T, Viertl D, et al (2010) Amyloid-beta aggregates cause alterations of astrocytic metabolic phenotype: impact on neuronal viability. J Neurosci 30: 3326–3338. 20203192 10.1523/JNEUROSCI.5098-09.2010 PMC6634099

[18] Glass CK, Saijo K, Winner B, Marchetto MC, Gage FH (2010) Mechanisms underlying inflammation in neurodegeneration. Cell 140: 918–934. 20303880 10.1016/j.cell.2010.02.016 PMC2873093

[19] Freir DB, Fedriani R, Scully D, Smith IM, Selkoe DJ, et al (2011) Abeta oligomers inhibit synapse remodelling necessary for memory consolidation. Neurobiol Aging 32: 2211–2218. 20097446 10.1016/j.neurobiolaging.2010.01.001 PMC2891223

[20] Shankar GM, Bloodgood BL, Townsend M, Walsh DM, Selkoe DJ, et al (2007) Natural oligomers of the Alzheimer amyloid-beta protein induce reversible synapse loss by modulating an NMDA-type glutamate receptor-dependent signaling pathway. J Neurosci 27: 2866–2875. 17360908 10.1523/JNEUROSCI.4970-06.2007 PMC6672572

[21] Shankar GM, Li S, Mehta TH, Garcia-Munoz A, Shepardson NE, et al (2008) Amyloid-beta protein dimers isolated directly from Alzheimer’s brains impair synaptic plasticity and memory. Nat Med 14: 837–842. 18568035 10.1038/nm1782 PMC2772133

[22] Cleary JP, Walsh DM, Hofmeister JJ, Shankar GM, Kuskowski MA, et al (2005) Natural oligomers of the amyloid-beta protein specifically disrupt cognitive function. Nat Neurosci 8: 79–84. 15608634 10.1038/nn1372

[23] Davalos AR, Coppe JP, Campisi J, Desprez PY (2010) Senescent cells as a source of inflammatory factors for tumor progression. Cancer Metastasis Rev 29: 273–283. 20390322 10.1007/s10555-010-9220-9 PMC2865636

[24] Maggio M, Guralnik JM, Longo DL, Ferrucci L (2006) Interleukin-6 in aging and chronic disease: a magnificent pathway. J Gerontol A Biol Sci Med Sci 61: 575–584. 16799139 10.1093/gerona/61.6.575 PMC2645627

[25] Freund A, Orjalo AV, Desprez PY, Campisi J (2010) Inflammatory networks during cellular senescence: causes and consequences. Trends Mol Med 16: 238–246. 20444648 10.1016/j.molmed.2010.03.003 PMC2879478

[26] Heneka MT, O’Banion MK (2007) Inflammatory processes in Alzheimer’s disease. J Neuroimmunol 184: 69–91. 17222916 10.1016/j.jneuroim.2006.11.017

[27] Ressler S, Bartkova J, Niederegger H, Bartek J, Scharffetter-Kochanek K, et al (2006) p16INK4A is a robust in vivo biomarker of cellular aging in human skin. Aging Cell 5: 379–389. 16911562 10.1111/j.1474-9726.2006.00231.x

[28] Krishnamurthy J, Torrice C, Ramsey MR, Kovalev GI, Al-Regaiey K, et al (2004) Ink4a/Arf expression is a biomarker of aging. J Clin Invest 114: 1299–1307. 15520862 10.1172/JCI22475 PMC524230

[29] Association As (2012) Alzheimer’s disease facts and figures. Alzheimer’s and Dementia: The Journal of the Alzheimer’s Association 8: 131–168. 10.1016/j.jalz.2012.02.001 22404854

[30] Livney MG, Clark CM, Karlawish JH, Cartmell S, Negron M, et al (2011) Ethnoracial differences in the clinical characteristics of Alzheimer’s disease at initial presentation at an urban Alzheimer’s disease center. Am J Geriatr Psychiatry 19: 430–439. 21522051 10.1097/JGP.0b013e3181f7d881 PMC3085004

[31] Hebert LE, Scherr PA, Bienias JL, Bennett DA, Evans DA (2003) Alzheimer disease in the US population: prevalence estimates using the 2000 census. Arch Neurol 60: 1119–1122. 12925369 10.1001/archneur.60.8.1119

[32] Chkhotua AB, Gabusi E, Altimari A, D’Errico A, Yakubovich M, et al (2003) Increased expression of p16(INK4a) and p27(Kip1) cyclin-dependent kinase inhibitor genes in aging human kidney and chronic allograft nephropathy. Am J Kidney Dis 41: 1303–1313. 12776284 10.1016/s0272-6386(03)00363-9

[33] Tsuji T, Aoshiba K, Nagai A (2006) Alveolar cell senescence in patients with pulmonary emphysema. Am J Respir Crit Care Med 174: 886–893. 16888288 10.1164/rccm.200509-1374OC

[34] Chimenti C, Kajstura J, Torella D, Urbanek K, Heleniak H, et al (2003) Senescence and death of primitive cells and myocytes lead to premature cardiac aging and heart failure. Circ Res 93: 604–613. 12958145 10.1161/01.RES.0000093985.76901.AF

[35] Wang HY, D’Andrea MR, Nagele RG (2002) Cerebellar diffuse amyloid plaques are derived from dendritic Abeta42 accumulations in Purkinje cells. Neurobiol Aging 23: 213–223. 11804705 10.1016/s0197-4580(01)00279-2

[36] Li YT, Woodruff-Pak DS, Trojanowski JQ (1994) Amyloid plaques in cerebellar cortex and the integrity of Purkinje cell dendrites. Neurobiol Aging 15: 1–9. 8159255 10.1016/0197-4580(94)90139-2

[37] West MD, Pereira-Smith OM, Smith JR (1989) Replicative senescence of human skin fibroblasts correlates with a loss of regulation and overexpression of collagenase activity. Exp Cell Res 184: 138–147. 2551704 10.1016/0014-4827(89)90372-8

[38] Coppe JP, Rodier F, Patil CK, Freund A, Desprez PY, et al (2011) Tumor suppressor and aging biomarker p16(INK4a) induces cellular senescence without the associated inflammatory secretory phenotype. J Biol Chem 286: 36396–36403. 21880712 10.1074/jbc.M111.257071 PMC3196093

[39] Iwasa H, Han J, Ishikawa F (2003) Mitogen-activated protein kinase p38 defines the common senescence-signalling pathway. Genes Cells 8: 131–144. 12581156 10.1046/j.1365-2443.2003.00620.x

[40] Freund A, Patil CK, Campisi J (2011) p38MAPK is a novel DNA damage response-independent regulator of the senescence-associated secretory phenotype. EMBO J 30: 1536–1548. 21399611 10.1038/emboj.2011.69 PMC3102277

[41] Bachstetter AD, Xing B, de Almeida L, Dimayuga ER, Watterson DM, et al (2011) Microglial p38alpha MAPK is a key regulator of proinflammatory cytokine up-regulation induced by toll-like receptor (TLR) ligands or beta-amyloid (Abeta). J Neuroinflammation 8: 79. 21733175 10.1186/1742-2094-8-79 PMC3142505

[42] Munoz L, Ammit AJ (2010) Targeting p38 MAPK pathway for the treatment of Alzheimer’s disease. Neuropharmacology 58: 561–568. 19951717 10.1016/j.neuropharm.2009.11.010

[43] Myung NH, Zhu X, Kruman, II, Castellani RJ, Petersen RB, et al (2008) Evidence of DNA damage in Alzheimer disease: phosphorylation of histone H2AX in astrocytes. Age (Dordr) 30: 209–215. 19424844 10.1007/s11357-008-9050-7 PMC2585649

[44] Ahronheim JC, Bernholc AS, Clark WD (1983) Age trends in autopsy rates. Striking decline in late life. JAMA 250: 1182–1186. 6876357

[45] Coppe JP, Patil CK, Rodier F, Sun Y, Munoz DP, et al (2008) Senescence-associated secretory phenotypes reveal cell-nonautonomous functions of oncogenic RAS and the p53 tumor suppressor. PLoS Biol 6: 2853–2868. 19053174 10.1371/journal.pbio.0060301 PMC2592359

[46] Tresini M, Mawal-Dewan M, Cristofalo VJ, Sell C (1998) A phosphatidylinositol 3-kinase inhibitor induces a senescent-like growth arrest in human diploid fibroblasts. Cancer Res 58: 1–4. 9426047

[47] Zeng G, Millis AJ (1996) Differential regulation of collagenase and stromelysin mRNA in late passage cultures of human fibroblasts. Exp Cell Res 222: 150–156. 8549657 10.1006/excr.1996.0019

[48] Golde TE, Miller VM (2009) Proteinopathy-induced neuronal senescence: a hypothesis for brain failure in Alzheimer’s and other neurodegenerative diseases. Alzheimers Res Ther 1: 5. 19822029 10.1186/alzrt5 PMC2874257

[49] Donnini S, Solito R, Cetti E, Corti F, Giachetti A, et al (2010) Abeta peptides accelerate the senescence of endothelial cells in vitro and in vivo, impairing angiogenesis. FASEB J 24: 2385–2395. 20207941 10.1096/fj.09-146456

[50] McLean CA, Cherny RA, Fraser FW, Fuller SJ, Smith MJ, et al (1999) Soluble pool of Abeta amyloid as a determinant of severity of neurodegeneration in Alzheimer’s disease. Ann Neurol 46: 860–866. 10589538 10.1002/1531-8249(199912)46:6<860::aid-ana8>3.0.co;2-m

[51] Hu J, Akama KT, Krafft GA, Chromy BA, Van Eldik LJ (1998) Amyloid-beta peptide activates cultured astrocytes: morphological alterations, cytokine induction and nitric oxide release. Brain Res 785: 195–206. 9518610 10.1016/s0006-8993(97)01318-8

[52] Hou L, Liu Y, Wang X, Ma H, He J, et al (2011) The effects of amyloid-beta42 oligomer on the proliferation and activation of astrocytes in vitro. In Vitro Cell Dev Biol Anim 47: 573–580. 21858610 10.1007/s11626-011-9439-y

[53] Walsh DM, Klyubin I, Fadeeva JV, Cullen WK, Anwyl R, et al (2002) Naturally secreted oligomers of amyloid beta protein potently inhibit hippocampal long-term potentiation in vivo. Nature 416: 535–539. 11932745 10.1038/416535a

[54] Verkhratsky A, Sofroniew MV, Messing A, Delanerolle NC, Rempe D, et al. (2012) Neurological diseases as primary gliopathies: A reassessment of neurocentrism. ASN Neuro. 10.1042/AN20120010 PMC3320215 22339481

[55] Hamby ME, Sofroniew MV (2010) Reactive astrocytes as therapeutic targets for CNS disorders. Neurotherapeutics 7: 494–506. 20880511 10.1016/j.nurt.2010.07.003 PMC2952540

[56] Fa M, Orozco IJ, Francis YI, Saeed F, Gong Y, et al. (2010) Preparation of oligomeric beta-amyloid 1–42 and induction of synaptic plasticity impairment on hippocampal slices. J Vis Exp. 10.3791/1884 PMC3156071 20644518

[57] Stine WB Jr, Dahlgren KN, Krafft GA, LaDu MJ (2003) In vitro characterization of conditions for amyloid-beta peptide oligomerization and fibrillogenesis. J Biol Chem 278: 11612–11622. 12499373 10.1074/jbc.M210207200

[58] Koo EH, Squazzo SL (1994) Evidence that production and release of amyloid beta-protein involves the endocytic pathway. J Biol Chem 269: 17386–17389. 8021238

[59] Walsh DM, Tseng BP, Rydel RE, Podlisny MB, Selkoe DJ (2000) The oligomerization of amyloid beta-protein begins intracellularly in cells derived from human brain. Biochemistry 39: 10831–10839. 10978169 10.1021/bi001048s

[60] Poling A, Morgan-Paisley K, Panos JJ, Kim EM, O’Hare E, et al (2008) Oligomers of the amyloid-beta protein disrupt working memory: confirmation with two behavioral procedures. Behav Brain Res 193: 230–234. 18585407 10.1016/j.bbr.2008.06.001 PMC2786170

[61] Puck TT, Cieciura SJ, Robinson A (1958) Genetics of somatic mammalian cells. III. Long-term cultivation of euploid cells from human and animal subjects. J Exp Med 108: 945–956. 13598821 10.1084/jem.108.6.945 PMC2136918

[62] Dimri GP, Lee X, Basile G, Acosta M, Scott G, et al (1995) A biomarker that identifies senescent human cells in culture and in aging skin in vivo. Proc Natl Acad Sci U S A 92: 9363–9367. 7568133 10.1073/pnas.92.20.9363 PMC40985

