# Supplementary material for: Astrocyte Senescence as a Component of Alzheimer’s Disease
Source: PLoS One. 2012 Sep 12;7(9):e45069. doi: 10.1371/journal.pone.0045069 (PMC3440417; doi:10.1371/journal.pone.0045069)
Supplement: Table S1 — Clinical history obtained from post-mortem reports. A total of 44 cases (4 fetal and 40 adults) were included in the study. Adult ages ranged from 35 to 92 years (mean 72.3±17.4; 10 males, 15 females) for controls and 49 to 90 years (mean 77.6±11.44; 8 males, 7 females) for patients with AD. The average postmortem interval was 13.31±9.44 hours (N = 36, range 3 to 43.5 hours). Fetal samples were obtained from fetuses that died in utero and therefore had uncertain postmortem intervals. Cortical sections were obtained from the frontal cortex in all the adult cases. (DOCX) [file pone.0045069.s001.docx]

**Supplementary Table 1:**

|  | Non-AD Controls (%) | AD (%) |
| --- | --- | --- |
| Braak and Braak |  |  |
| I/II | 4/21 (19) |  |
| II/III | 1/21(4.7) |  |
| V/VI |  | 11/15 (73.3) |
| Family history of AD | 0/21 (0) | 3/15 (20) |
| Clinical dementia | 1/21(4.7) | 15/15 (100) |
| Hypertension | 9/17(52.9) | 4/11 (36.4) |
| Diabetes mellitus | 5/17(29.4) | 3/14 (21.4) |
| Chronic obstructive pulmonary disease | 8/17 (47.1) | 2/14 (14.3) |
| Smoker | 5/17 (29.4) | 6/14 (42.9) |
| Perimortem hypoxic events | 14/14 (100) | 4/4 (100) |
| Pathologic evidence of CNS hypoxia | 15/19*(78.9) 4/19**(21.1) | 1/14(7.1) |

* Absent

** Evidence of remote hypoxic event
