# Supplementary figures and images for: Astrocyte Senescence as a Component of Alzheimer’s Disease
Source: PLoS One. 2012 Sep 12;7(9):e45069. doi: 10.1371/journal.pone.0045069 (PMC3440417; doi:10.1371/journal.pone.0045069)

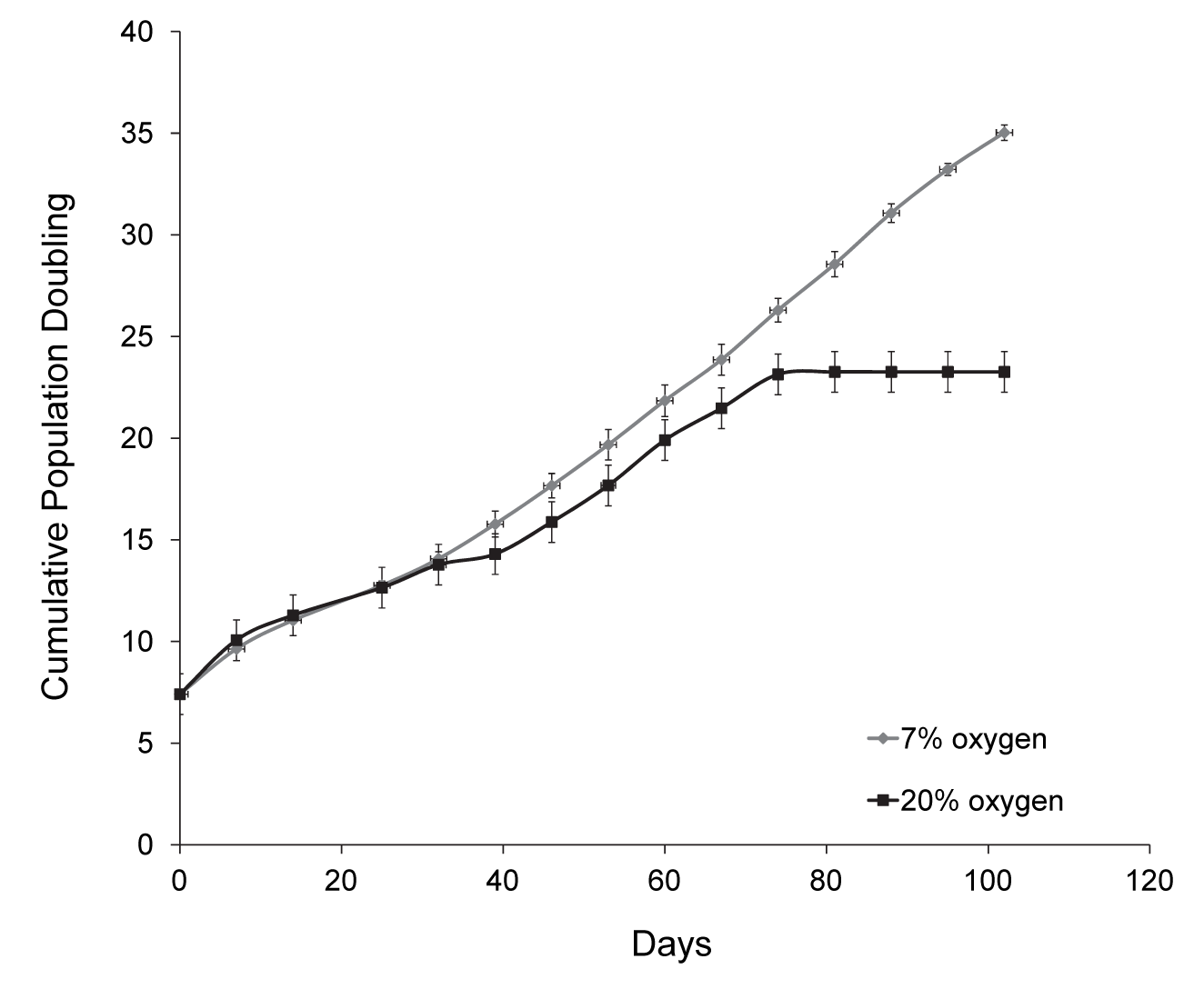

Supplement: Figure S1 — Effect of oxygen tension on replicative lifespan. Astrocytes were grown at 5% CO2 and either 20% or 7% oxygen. At every passage, cells were counted and the cumulative population doubling was calculated as described [13]. (TIF) [file pone.0045069.s002.tif]

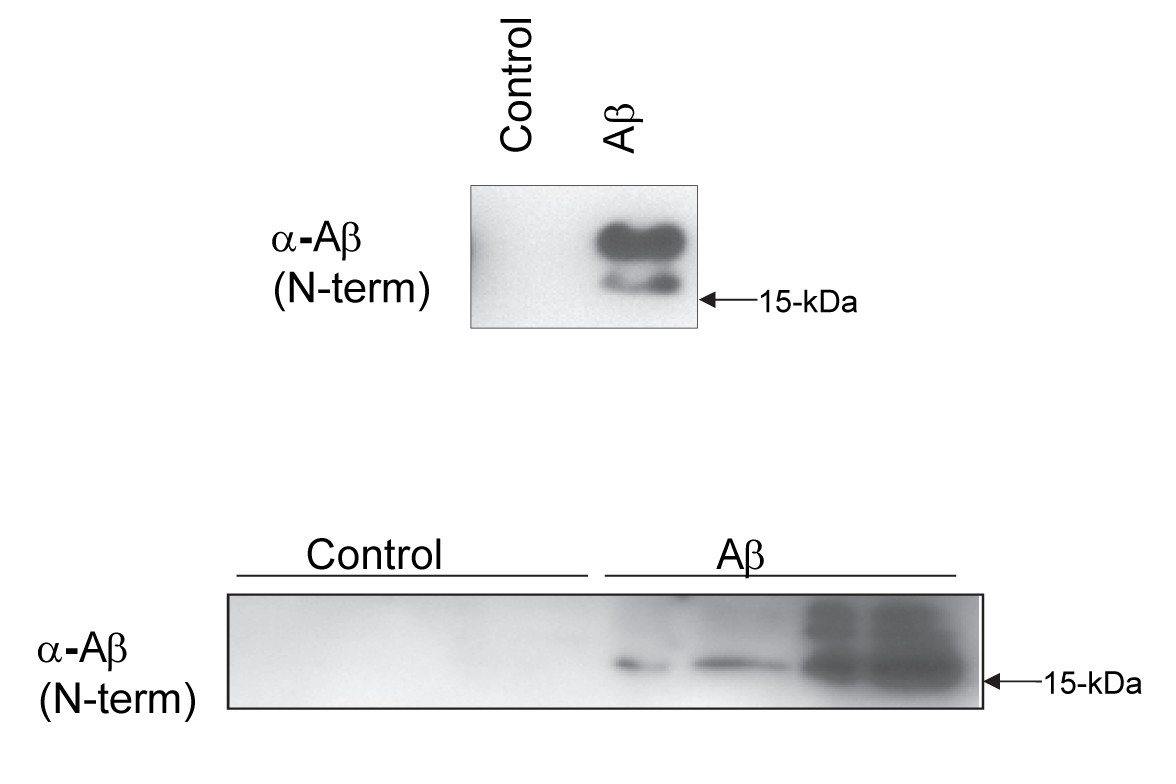

Supplement: Figure S2 — Detection of oligomerized Aβ peptide. Top gel, synthetic amyloid-β peptide (Aβ1–42) was diluted in astrocyte media to obtain a final concentration of 1µM and oligomerized as described. Astrocyte media with dimethyl sulfoxide (DMSO) alone was used as a control. A volume of media containing approximately 0.094 µg of synthetic peptide was loaded onto 12% gel. Western blot depicts the presence of small molecular weight oligomers of Aβ in astrocyte media. Bottom gel, conditioned media from 7PA2 cells contains secreted Aβ. 7PA2 and control CHO cells were incubated with serum-free DMEM or MCDB105 media for 24 hours to generate conditioned media. Conditioned media was collected and concentrated as described. Western blot showing presence of Aβ in conditioned media from 7PA2 cells. (TIF) [file pone.0045069.s003.tif]
